# Temperature-Dependent Effects of Cutaneous Bacteria on a Frog’s Tolerance of Fungal Infection

**DOI:** 10.3389/fmicb.2018.00410

**Published:** 2018-03-07

**Authors:** Matthew J. Robak, Corinne L. Richards-Zawacki

**Affiliations:** ^1^Department of Ecology and Evolutionary Biology, Tulane University, New Orleans, LA, United States; ^2^Department of Biological Sciences, University of Pittsburgh, Pittsburgh, PA, United States

**Keywords:** *Acris crepitans*, amphibian chytridiomycosis, antifungal, bioaugmentation, host–pathogen interactions, skin microbes, *Stenotrophomonas maltophilia*

## Abstract

Defense against pathogens is one of many benefits that bacteria provide to animal hosts. A clearer understanding of how changes in the environment affect the interactions between animals and their microbial benefactors is needed in order to predict the impact and dynamics of emerging animal diseases. Due to its dramatic effects on the physiology of animals and their pathogens, temperature may be a key variable modulating the level of protection that beneficial bacteria provide to their animal hosts. Here we investigate how temperature and the makeup of the skin microbial community affect the susceptibility of amphibian hosts to infection by *Batrachochytrium dendrobatidis (Bd)*, one of two fungal pathogens known to cause the disease chytridiomycosis. To do this, we manipulated the skin bacterial communities of susceptible hosts, northern cricket frogs (*Acris crepitans*), prior to exposing these animals to *Bd* under two different ecologically relevant temperatures. Our manipulations included one treatment where antibiotics were used to reduce the skin bacterial community, one where the bacterial community was augmented with the antifungal bacterium, *Stenotrophomonas maltophilia*, and one in which the frog’s skin bacterial community was left intact. We predicted that frogs with reduced skin bacterial communities would be more susceptible (i.e., less resistant to and/or tolerant of *Bd* infection), and frogs with skin bacterial communities augmented with the known antifungal bacterium would be less susceptible to *Bd* infection and chytridiomycosis. However, we also predicted that this interaction would be temperature dependent. We found a strong effect of temperature but not of skin microbial treatment on the probability and intensity of infection in *Bd*-exposed frogs. Whether temperature affected survival; however, it differed among our skin microbial treatment groups, with animals having more *S. maltophilia* on their skin surviving longer at 14 but not at 26°C. Our results suggest that temperature was the predominant factor influencing *Bd*’s ability to colonize the host (i.e., resistance) but that the composition of the cutaneous bacterial community was important in modulating the host’s ability to survive (i.e., tolerate) a heavy *Bd* infection.

## Introduction

That there exist mutualistic and even symbiotic relationships between animals and microbes has long been understood, yet it is only recently that we have come to appreciate how common and influential these relationships can be for ecological processes that play out across taxa and environments (reviewed in McFall-Ngai et al., 2013). While effects of temperature on microbial growth and community structure in soil and other environmental samples have been well-documented (e.g., Jansson and Tas, 2014; Piquet et al., 2016), how temperature variation affects animal–microbe interactions is less well-understood. Empirical data exist for a few hosts and their bacterial symbionts (e.g., sponges: Webster et al., 2008; Fan et al., 2013; sea anemone: Fraune et al., 2016), yet the range of taxa and types of interactions under which this relationship with temperature has been explored remains small (Carey and Duddleston, 2014). Studies that broaden this range of hosts and ecological contexts are needed to clarify how temperature affects animal interactions with microbes, and how these effects may impact wildlife responses to environmental stressors such as climate change and habitat modification.

Defense against infection is one important service that microbes can provide for their animal hosts (McFall-Ngai et al., 2013; Clavel et al., 2017) and changes in temperature can affect the potential of microbes to cause disease or help their hosts resist infections (Daskin and Alford, 2012). For example, elevated ocean temperatures increase the expression of virulence genes in the bacterial pathogen *Vibrio shiloi*, which induces bleaching in the coral *Oculina patagonica* (Rosenberg and Ben-Halm, 2002). On the flip side, the ability of ascidians to defend themselves against pathogens is likely impacted by effects of temperature on their community of symbiotic microbes (Tianero et al., 2015); bacterial symbionts provide these animals with their diverse repertoire of defensive secondary metabolites, some of which have antimicrobial and antiviral properties (Paul et al., 1990). Temperature may be a key player in determining the health benefits that symbiotic microbes bestow upon their animal hosts, especially for ectotherms (Kohl and Yahn, 2016; Ferguson, 2017), though clear empirical examples appear to be limited to handful of invertebrates (Tianero et al., 2015; Ferguson, 2017). Amphibians are another taxon useful for investigating the effects of temperature on microbial symbioses, as several aspects of the amphibian immune system are known to function in a temperature-dependent manner (Maniero and Carey, 1997; Rollins-Smith and Woodhams, 2012) and the cutaneous bacteria of amphibians are known to be important to their defense against other skin microbes, including pathogenic fungi in the genus *Batrachochytrium* (Harris et al., 2009a).

Chytridiomycosis, the disease caused by the chytrid fungi *Batrachochytrium dendrobatidis* (*Bd*) and *Batrachochytrium salamandrivorans*, has been implicated in global amphibian declines (Berger et al., 1998; Lips et al., 2006; Rachowicz et al., 2006; Martel et al., 2013). Because *Batrachochytrium salamandrivorans* has only recently been described (Martel et al., 2013), less is known about its potential consequences for host populations (but see Martel et al., 2014). *Bd*, however, is known to disrupt electrolyte transport across frog skin, which can cause cardiac arrest, the mechanism of mortality (Voyles et al., 2009). Not all amphibians are equally at risk of infection. Hosts found in consistently cool, wet habitats in both temperate and tropical regions appear particularly vulnerable to *Bd*-related declines (Berger et al., 2016). *Bd* infection dynamics have also been correlated with climate and seasonality (Berger et al., 2004; Woodhams and Alford, 2005; Bishop et al., 2009; Rohr and Raffel, 2010), with infections often peaking in early spring (Kriger and Hero, 2007; Longcore et al., 2007; Rothermel et al., 2008). Variation in susceptibility to chytridiomycosis also exists within and among host species (Tobler and Schmidt, 2010; Martel et al., 2014), with some species requiring a higher pathogen load in order to become sick than others (Berger et al., 2004). This could be caused by differences among strains of *Bd*, as some are more pathogenic than others (Retallick and Miera, 2007; Farrer et al., 2011). Differences in susceptibility among hosts (Woodhams et al., 2007a) and populations (Savage and Zamudio, 2011) could also reflect intrinsic or temperature-driven differences in host immunity (reviewed in Rowley and Alford, 2010; Rollins-Smith and Woodhams, 2012).

Amphibian hosts have several potential lines of defense against *Batrachochytrium* pathogens. Antimicrobial peptides (AMPs), produced in the granular glands of the skin of some amphibians and secreted in mucus, have been shown to inhibit the growth of *Bd in vitro* (Rollins-Smith et al., 2006; Ramsey et al., 2010). Antibody and lymphocyte production is also stimulated by *Bd* exposure in some host species, suggesting the potential for an acquired immune response to this pathogen (Ramsey et al., 2010; McMahon et al., 2014). Variation in body temperature among individuals has also been correlated with the probability of *Bd* infection (Richards-Zawacki, 2010; Rowley and Alford, 2013; Roznik et al., 2015). Amphibians may also elevate their body temperature above normal by selecting warmer microhabitats, thereby inducing a behavioral fever (Sherman and Stephens, 1998; Woodhams et al., 2003; Sherman, 2008; Richards-Zawacki, 2010). This elevated temperature presumably enhances the immune response (Maniero and Carey, 1997; Rollins-Smith and Woodhams, 2012) though the effectiveness of such a fever in combatting *Bd* infection has not been demonstrated empirically.

Another component of amphibian defense against pathogens is the skin microbiome. The mucus on frog skin is home to a rich community of bacteria (McKenzie et al., 2012; Kueneman et al., 2013). While there is also variation within frog species (Lauer et al., 2008), cutaneous bacterial communities have been found to differ more among amphibian species than among individuals and/or environments (McKenzie et al., 2012; Becker et al., 2014). This suggests that innate differences in skin bacterial communities among species could contribute to differences in susceptibility to chytridiomycosis (McKenzie et al., 2012). Some members of amphibian skin bacterial communities are known to produce substances with antifungal capabilities. This was first demonstrated in two North American salamander species by Harris et al. (2006), who isolated skin bacteria and challenged *Bd* to grow in the presence of those isolates *in vitro*. Since then, a large and growing number of bacteria found on amphibian skin have been shown to inhibit the growth of *Bd* (Woodhams et al., 2015). It is believed to be metabolites produced by these bacteria that inhibit *Bd* growth and confer resistance and/or tolerance to infection in amphibian hosts (Brucker et al., 2008a,b; Harris et al., 2009a; Becker et al., 2015). Support for this idea comes from *in vitro* studies where the metabolites produced by a variety of bacteria isolated from frog mucus inhibit the growth of *Bd* (Daskin et al., 2014; Woodhams et al., 2015).

Laboratory exposure studies provide further evidence that bacteria found on frog skin can contribute to variation in susceptibility to chytridiomycosis (Harris et al., 2009b; Woodhams et al., 2014). For example, adding *Janthinobacterium lividum*, a bacterium that produces the *Bd*-inhibitory metabolite violacein, to the skin of *R. muscosa* decreased the risk of mortality after *Bd* exposure (Harris et al., 2009a). Correlations between skin bacterial communities and susceptibility to chytridiomycosis have been documented in wild populations as well (Woodhams et al., 2007b). Given this, efforts are underway to develop probiotics that could be applied to frogs to protect them from *Bd* in the wild (Bletz et al., 2013). Probiotic approaches may prove to be the most plausible solution for *in situ* conservation of species that are threatened with extinction due to *Bd*, but their effectiveness may be dependent upon environmental conditions, including temperature (Woodhams et al., 2014).

Temperature is known to affect the growth (Gaddad and Rodgi, 1987; Pietikainen et al., 2005) and antifungal metabolite production (Noaman et al., 2004; Ripa et al., 2009; Kariluoto et al., 2010) of bacteria. There is also some evidence that the bacteria found on frog skin produce anti-*Bd* metabolites better at some temperatures than others. Daskin et al. (2014) found that the cell free supernatants from frog skin bacteria cultured at cooler temperatures were less effective at inhibiting the growth of *Bd in vitro*. Robak (2016) also found that antifungal metabolites produced by frog skin bacteria were more effective at inhibiting *Bd* growth at higher temperatures, but in this study, the temperature at which the metabolites were produced was less important than the temperature at which the growth challenge assay was performed. If this temperature dependence of anti-*Bd* activity is a general phenomenon, it could contribute to the observed correlations between frog body temperatures and *Bd* infection (Richards-Zawacki, 2010; Rowley and Alford, 2013; Roznik et al., 2015) and between climatic variation and chytridiomycosis (Berger et al., 2004; Woodhams and Alford, 2005; Rohr and Raffel, 2010). It also suggests that development of a successful probiotic treatment for *Bd* infected animals will require information on how the chosen bacteria would function in natural environments, where conditions such as temperature vary in space and time. In the Robak (2016) study, one bacterium of interest, *Stenotrophomonas maltophilia*, was found to produce metabolites that inhibit the growth of *Bd in vitro* at 14, 20, and 26°C, although the extent of inhibition was greatest at 20°C. While its products effectively inhibit *Bd* growth across a range of ecologically relevant temperatures in culture, it is not known whether (1) *S. maltophilia* presence on the skin would protect frogs from *Bd* infection and/or chytridiomycosis, or whether (2) the relationship between temperature and protection on hosts would mirror what was seen for *Bd* growth *in vitro*.

In this study, we examined the effect of temperature on the ability of skin microbes to protect an amphibian host against *Bd* infection and chytridiomycosis. To do this, we manipulated the skin bacterial communities of northern cricket frogs (*Acris crepitans*), a species known to be susceptible to chytridiomycosis (Zippel and Tabaka, 2008; Sonn et al., 2017) and either exposed them to *Bd* or sham-exposed them and housed half of each group at 14°C and the other half at 26°C. These temperatures were chosen as they are within the range of body temperatures that this host experiences during times of the year when they are infected with *Bd* in the wild (Sonn, 2016) and because susceptibility to chytridiomycosis in this host has been shown to differ between these two temperatures (Sonn et al., 2017). Within each temperature and exposure group (*Bd* vs. sham), frog skin bacterial communities were either (1) maintained intact, (2) reduced with antibiotics, or (3) augmented by inoculation with *S. maltophilia* (family Xanthomonadaceae, order Xanthomonadales). This Gram-negative bacterium found on frog skin, as well as in water, soil, and plant samples from a wide variety of environments and geographic regions (Denton and Kerr, 1998), has been demonstrated to inhibit *Bd* growth *in vitro* (Robak, 2016). We predicted that *Bd*-exposed frogs at the lower temperature would be more susceptible to chytridiomycosis, which we defined as having a greater *Bd* load (Voyles et al., 2009), decreased survival (Voyles et al., 2009), a higher prevalence of *Bd* infection (Vredenburg et al., 2010), or a lower body condition (Retallick and Miera, 2007; Murphy et al., 2011). We also predicted that frogs with their bacterial communities reduced would be more susceptible, and frogs with *S. maltophilia* added would be less susceptible to chytridiomycosis than frogs with intact skin microbial communities (Harris et al., 2009a), but that the effect of *S. maltophilia* on *Bd* susceptibility would be temperature dependent.

## Materials and Methods

### Animal Husbandry

In February 2016, we collected 122 *A. crepitans* frogs from Tulane University’s F. Edward Hebert Riverside Research Center near Belle Chasse, LA, United States (WGS84: 29.8852489, -89.9694904) and placed them individually into cylindrical plastic enclosures (15 cm tall, 11 cm diameter with ventilated lids) containing a 2.5 cm depth of filtered tap water. While the previous infection history of these individual frogs was not known, *Bd* had been detected, sometimes at greater than 50% prevalence, in this population (Brannelly et al., unpublished data). To clear any potential *Bd* infections, we heat-treated the animals by holding them at 30°C in an environmental chamber (Conviron, Adaptis; 12 h light/dark cycle) for 10 days (Chatfield and Richards-Zawacki, 2011). Given the high prevalence of *Bd* in this population and our observation that individuals frequently gain and lose infections in the wild (Brannelly et al., unpublished data), we assumed that most or all of these animals were likely exposed to *Bd* prior to this study, and if any immunoprotective effects of prior-exposure existed, they did not preclude animals from becoming re-infected. However, our study design did not permit us to control for immunoprotective effects of prior *Bd*-exposure explicitly. After heat treatment, we tested the frogs for *Bd* following the swabbing and quantitative polymerase chain reaction (qPCR) assay protocols described below. After heat-treatment and during bacterial manipulations, we housed the frogs at 20°C. To get to this temperature, we lowered the temperature gradually over a period of 28 h. We assigned animals haphazardly to temperature, bacterial manipulation, and exposure groups, with each combination of temperature, bacterial manipulation, and exposure containing either 9, 10, or 11 animals. After bacterial manipulation, frogs were housed at either 14 or 26°C and were either inoculated with *Bd* or sham inoculated (**Table 1**). We fed the frogs *ad libitum* on 2 week-old crickets and provided them with a clean enclosure and fresh water every 7 days. We cleaned the enclosures with a 10% bleach solution and allowed them to dry completely before reuse. We wore a clean pair of nitrile gloves when handling each frog. We carried out this study in accordance with the recommendations of Tulane University’s Institutional Animal Care and Use Committee (IACUC, Protocol No. 0391R2).

**Table 1:** Treatment groups.

*n*	Bacteria	Inoculation	Temperature (°C)
10	Reduced	Sham	14
10	Reduced	Sham	26
10	Reduced	*Bd*	14
10	Reduced	*Bd*	26
9	Intact	Sham	14
10	Intact	sham	26
10	Intact	*Bd*	14
11	Intact	*Bd*	26
10	Added	sham	14
10	Added	sham	26
11	Added	*Bd*	14
11	Added	*Bd*	26

*n = number of frogs; bacteria = bacterial manipulation; inoculation = inoculated with *Bd* or sham inoculated; and temperature = temperature frogs were housed at after bacterial manipulations*.

### Animal Monitoring

We monitored frogs daily for the following clinical signs of chytridiomycosis: lethargy, inappetence, loss of righting reflex, excessive skin sloughing, abnormal posture, and cutaneous erythema (Berger et al., 2005). To test for *Bd* infection and the presence of *S. maltophilia* on frog skin, we rinsed the frogs in filtered tap water and then swabbed the skin by rubbing a rayon tipped swab (MWE 113, Medical Wire and Equipment, Co., United Kingdom) five times over the dorsum, venter, each side of the body, and the bottom of each foot. This was done once each week starting on day 6. Snout-vent length (SVL), measured to the nearest 0.1 mm with a dial calipers, and mass, measured with a scale to the nearest 0.01 g, were recorded weekly, starting on day -1, the day prior to the first round of *Bd* inoculations (**Table 2**). We used residual mass as our index of body condition and we calculated this using the line of best fit from a linear regression between SVL and body mass for all frogs on day -1 (Supplementary Figure S1). The predicted mass of each frog, based on this pre-experiment regression was then subtracted from the actual mass each week to get a residual value, which reflects body condition relative to the mean for a frog of that size prior to *Bd* exposure (Jakob et al., 2011). Frogs were euthanized by bath in tricaine methane sulfonate (MS-222, pH 7) at the conclusion of the experiment.

**Table 2:** Summary timeline for bacterial manipulations.

Day	Treatment
-5	Provosoli bath
-4	Antibiotic (BCR^∗^ and SMA) or filtered water (BCI) bath
-3	Fresh antibiotic (BCR and SMA) or filtered water (BCI) bath
-2	Filtered water (BCR), Provosoli bath (BCI), or Provosoli bath with added
	*S. maltophilia* (SMA)
-1	Returned to enclosure
0	*Bd* or sham exposures
1	Returned to enclosure

^∗^*Treatment groups: BCR, bacterial community reduced; SMA, *S. maltophilia* added; and BCI, bacterial community intact*.

### Skin Microbe Quantification

We extracted genomic DNA from skin swabs using the Qiagen DNeasy Blood and Tissue kit, following the protocol for animal tissues with two modifications: (1) we incubated swabs for just 1 h, vortexing and spinning them in a centrifuge after 30 and 60 min of incubation; (2) we eluted samples twice with 100 μL of elution buffer instead of once with 200 μL. We then used a qPCR assay, performed on an Applied Biosystems 7500 system, to quantify the amount of *Bd* [in plasmid equivalents (PEs)]. We followed the protocol of Boyle et al. (2004) with the following modifications: (1) 0.7 μL of bovine serum albumin (Applied Biosystems) was added to each well prior to amplification (Garland et al., 2010) and (2) a sevenfold dilution series of *Bd* plasmid standards (Pisces Molecular, Boulder, CO, United States) was included in each run. For *S. maltophilia*, we quantified colony forming units (cfus) per swab using the same qPCR reaction cycling conditions and reagent concentrations as for *Bd*, but with primers and probes from Rios-Licea et al. (2010). For *S. maltophilia*, we generated a sevenfold dilution series of cfu standards by making serial dilutions of DNA extracted from a sample containing 5 × 10^6^ cfus of *S. maltophilia*. We ran qPCRs on all swab samples in singlicate and considered animals positive for *Bd* if the qPCR result indicated that one or more copies of *Bd* DNA (i.e., ≥1 PE) were present in the reaction. Animals were considered to have become infected if they tested positive for *Bd* on one or more weekly swab samples. We converted *Bd* and *S. maltophilia* loads per 5 μL reaction volume to whole swab loads and then log-transformed these values prior to statistical analysis.

### Bacterial Manipulations

Our skin bacterial community manipulations took place over 4 days and ended on the day prior to the first *Bd* (or sham) exposure (see **Table 2**). On day -5, we placed frogs in a bath of 30 mL modified Provosoli medium (Wyngaard and Chinnappa, 1982) for 24 h to collect native cutaneous bacteria. On day -4, we removed the frogs from their Provosoli baths and placed these baths in a refrigerator. Animals in the “reduced” bacterial community and “*S. maltophilia* added” treatments were moved directly into a second bath, this time containing 30 mL of an antibiotic cocktail that targets both Gram-negative and Gram-positive bacteria (24 mg/L cephalexin, 14.5 mg/L sulfamethazine, 2.9 mg/L trimethoprim, 100 mg/L streptomycin, and 10^5^ I.U./L penicillin) for 24 h (following Holden et al., 2015). Frogs in the “intact” bacterial community treatment were instead moved into a bath containing 30 mL of filtered tap water for 24 h. On day -3, we placed the frogs into either a fresh antibiotic cocktail bath with the same composition as the first (“reduced” and “*S. maltophilia* added” treatments) or into a filtered water bath (“intact” treatment) for 24 h. On day -2, we rinsed all frogs with filtered tap water and frogs in the “reduced” treatment group were placed in a bath containing 30 mL of filtered water for 24 h. Frogs in the “intact” treatment were placed back in their Provosoli baths (from day -4). Frogs in the “*S. maltophilia* added” treatment were placed back in their Provosoli baths also, but only after adding 2 × 10^8^ *S. maltophilia* to the bath. We left the frogs in these baths for 24 h. On day -1, we rinsed all frogs with filtered tap water and placed them in clean enclosures.

### *Bd* Exposure

To prepare inoculum for *Bd* exposures, we grew *Bd* (JEL412 isolated from a *Sachatamia ilex* frog in Panama in 2005 and provided by Dr. Joyce Longcore) on 1% tryptone agar plates for 7 days, at which point we harvested zoospores by flooding plates with 5 mL of deionized water. We then exposed frogs individually by placing them in a bath of 2 × 10^6^ zoospores suspended in 30 mL filtered tap water for 12 h. We carried out sham exposures in the same way except that 1% tryptone plates without *Bd* were flooded. We exposed frogs in this way weekly throughout the experiment, starting on day 0.

### Data Analysis

We used generalized linear mixed models (GLMMs) to test for significant effects of bacterial manipulations, temperature, and an interaction between temperature and bacterial manipulation on the log-transformed *Bd* and S. *maltophilia* loads on *Bd*-exposed frogs. Our model contained fixed effects of temperature, bacterial community manipulation (“reduced,” “intact,” or “*S. maltophilia* added”), and the interaction between those variables with day as a repeated measure. For these and all following GLMMs, we used a first-order autoregressive covariance type for repeated effects and residuals, assumed a non-normal distribution, and used a Satterthwaite approximation for degrees of freedom. A second GLMM with these same fixed effect plus exposure group (*Bd*- vs. sham-exposed) allowed us to test for significant main and interactive effects of temperature, bacterial manipulation, and *Bd* exposure on body condition. We used a third GLMM to test whether temperature, bacterial treatment, or their interaction affected the probability that *Bd*-exposed frogs became infected (yes/no). This model used a binary logistic distribution and the events/trials syntax, with infection as the event and day as the trial. To test for significant differences in survival among treatments (bacterial manipulations, temperatures, and exposure groups), we used a Cox regression with log-transformed *Bd* load included as a covariate. For significant effects, we used a Kaplan–Meier survival analysis to compute Tarone–Ware pairwise comparisons among groups.

As an alternative analytical approach, since *S. maltophilia* loads did not always differ among our bacterial community manipulation groups (see section “Results”), we repeated the analyses for *Bd* load, body condition, probability of infection (yes/no), and survival described above, but this time replacing the fixed effect of bacterial community manipulation (“reduced,” “intact,” or “*S. maltophilia* added”) with the covariate log-transformed *S. maltophilia* load (as determined by qPCR). These models included only *Bd*-exposed animals. When we had significant interaction effects with temperature in our survival analysis, we used separate Cox regressions for each experimental temperature (with Bonferroni-corrected *p*-values) for *post hoc* comparisons since Kaplan–Meier survival analyses cannot handle continuous factors like *S. maltophilia* load. All analyses were performed in IBM SPSS Statistics (v 23).

## Results

*Stenotrophomonas maltophilia* was detected on all frogs from all three bacterial manipulation groups and both temperatures throughout the experiment, with the exception of two frogs at 26°C that each tested negative in one weekly sample: one frog from the “*S. maltophilia* added” group tested negative on day 41 and one from the “intact” bacteria group tested negative on day 55 (Supplementary Figures S2A,B). The amount of *S. maltophilia* on the skin differed among temperature (GLMM: *F*_1,239_ = 30.608, *P* < 0.001) and bacterial manipulation (GLMM: *F*_2,239_ = 5.538, *P* = 0.004) groups, but the interaction between temperature and bacterial manipulation was not significant (GLMM: *F*_2,239_ = 1.618, *P* = 0.200). Frogs at 14°C had greater *S. maltophilia* loads than frogs at 26°C and frogs in the “intact” bacterial treatment had lower *S. maltophilia* loads than frogs in the “reduced” and “*S. maltophilia* added” groups (Tukey LSD: *t*_239_ ≥ 2.960, *P* ≤ 0.003, Supplementary Figure S3). There was no significant difference in *S. maltophilia* load between frogs in the “reduced” and “*S. maltophilia* added” treatments (Tukey LSD: *t*_239_ = 0.047, *P* = 0.962), suggesting that our cocktail of antibiotics did not reduce *S. maltophilia* abundance on frog skin.

**FIGURE 1:**
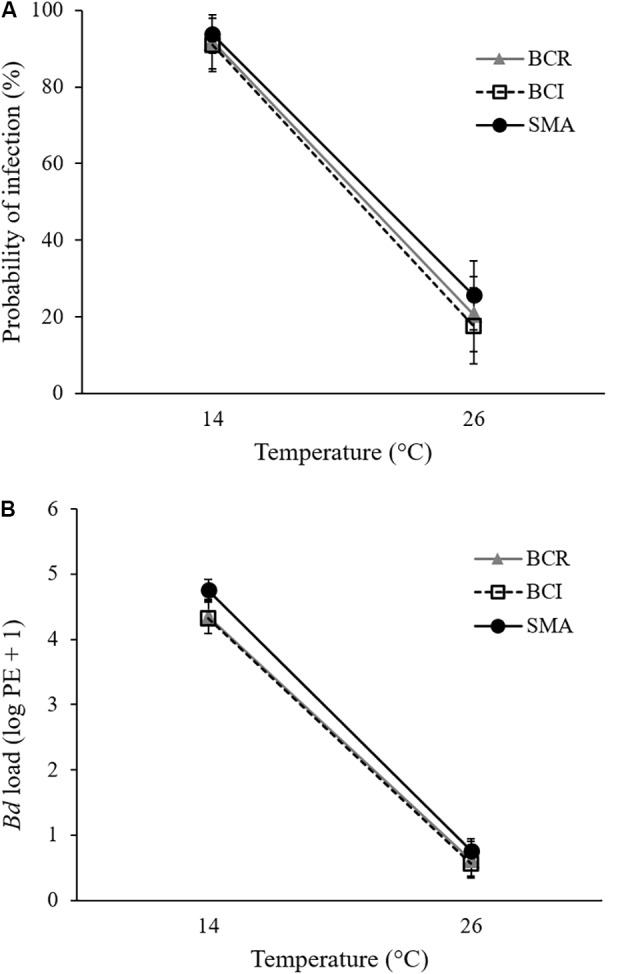
Mean (± SE) of **(A)** probability of infection and **(B)** *Bd* load, measured as log_10_ of plasmid equivalents (PEs) for *Bd*-exposed frogs at two treatment temperatures and three bacterial manipulations (BCR, bacterial community reduced; BCI, bacterial community intact; and SMA, *S. maltophilia* added) over the 9 weeks of the experiment.

All animals in *Bd*-exposed treatment groups had a positive qPCR result for *Bd* on at least one week of the experiment (Supplementary Figures S2C,D) and were therefore considered to have become infected. However, at 26°C, our *Bd*-exposed frogs tended to test positive for *Bd* only once or twice during the initial weeks of the experiment and then clear their infections. None of the sham-exposed animals ever tested positive for *Bd*. *Bd*-exposed frogs at 14°C had a greater probability of testing positive for *Bd* on any given swab than did *Bd*-exposed frogs at 26°C (GLMM: *F*_1,106_ = 90.731, *P* < 0.001; **Figure 1A**). There was no significant effect of bacterial manipulation (GLMM: *F*_2,106_ = 0.097, *P* = 0.908) and no significant interaction between temperature and bacterial manipulation (GLMM: *F*_2,106_ = 0.151, *P* = 0.860) on the weekly probability of *Bd* infection. When *S. maltophilia* load replaced bacterial manipulation in our model, the result was similar: neither *S. maltophilia* load (GLMM: *F*_1,184_ = 1.174, *P* = 0.280) nor the interaction between *S. maltophilia* load and temperature (GLMM: *F*_1,193_ = 1.528, *P* = 0.218) was significant predictors of *Bd* infection in weekly swab samples.

**FIGURE 2:**
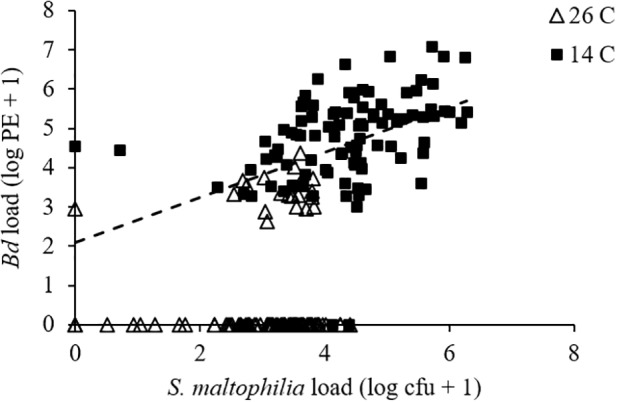
Relationship between *Bd* load, measured as log_10_ of PEs and *S. maltophilia* load, measured as log_10_ of colony forming units (cfus) for *Bd*-exposed frogs. Line is the least-squares line of best fit (*y* = 0.5722*x* + 2.0977; *R*^2^ = 0.171) for frogs at 14°C. The relationship was non-significant at 26°C.

For pathogen load, we found a significant effect of temperature (GLMM: *F*_1,175_ = 446.463, *P* < 0.001) but not of bacterial manipulation (GLMM: *F*_2,190_ = 1.521, *P* = 0.221) and the interaction between temperature and bacterial manipulation was not significant (GLMM: *F*_2,190_ = 0.254, *P* = 0.776). *Bd*-exposed animals at 14°C had greater *Bd* loads than those at 26°C (**Figure 1B** and Supplementary Figures S2C,D). When *S. maltophilia* load replaced bacterial manipulation in our model, the effect of temperature remained significant (GLMM: *F*_1,197_ = 4.659, *P* = 0.032) but the main effect of *S. maltophilia* load (GLMM: *F*_1,188_ = 4.559, *P* = 0.034) and the interaction between *S. maltophilia* load and temperature (GLMM: *F*_1,186_ = 6.924, *P* = 0.009) were also significant. *Post hoc* linear regressions (**Figure 2**) showed that the relationship between *S. maltophilia* load and *Bd* load on swabs was positive at 14°C (*R*^2^ = 0.171, *B* = 0.572, *F*_1,99_ = 20.466, *P* < 0.001) but non-significant at 26°C (*R*^2^ = 0.003, *F*_1,100_ = 0.350, *P* = 0.555).

Neither temperature (GLMM: *F*_1,672_ = 2.827, *P* = 0.093), bacterial manipulation (GLMM: *F*_1,672_ = 0.212, *P* = 0.809), nor exposure group (*Bd* vs. sham, GLMM: *F*_1,672_ = 0.083, *P* = 0.773) had a significant effect on body condition and there were no significant two- or three-way interactions between temperature, bacterial manipulation, and exposure group (GLMM: *F*_2,672_ ≤ 2.483, *P* ≥ 0.084). Results were similar when *S. maltophilia* load replaced bacterial manipulation in our model (GLMM: all *F*_1,201_ ≤ 1.702, all *P* ≥ 0.194). Body condition did change over the course of the experiment, though (GLMM, day: *F*_9,672_ = 1.950, *P* = 0.043; Supplementary Figure S4), with frogs increasing in body condition during the initial 3 weeks of the experiment (Tukey HSD: all pairwise *t*_672_ ≤ 1.834, *P* ≥ 0.05 except for day -1 vs. days 6, 13, and 20, which had *t*_672_ ≥ 2.464, *P* ≤ 0.014).

Clinical signs of chytridiomycosis and mortality were observed in *Bd*-exposed animals beginning 8 days after the initial *Bd* exposure. By the end of the experiment, 62 days after the first exposure, only five *Bd*-exposed frogs survived. Four of these were from 26°C treatments (one from each of the “intact” and “reduced” bacterial treatments and two from the “*S. maltophilia* added” treatment). The only surviving frog at 14°C was from the “reduced” bacterial community treatment (Supplementary Figures S2E,F). Mortality was low in sham-infected frogs (≤2 deaths per group), no clinical signs of chytridiomycosis were observed, and there were no significant differences in survival with respect to temperature or bacterial treatment (Tarone–Ware: *X*_2_ ≤ 1.108, *P* ≥ 0.293).

**FIGURE 3:**
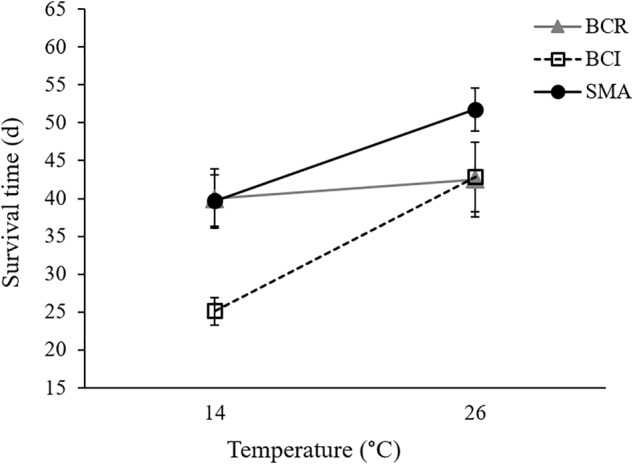
Mean (± SE) survival time for *Bd*-exposed frogs in three bacterial manipulation groups (BCR, bacterial community reduced; BCI, bacterial community intact; and SMA, *S. maltophilia* added) at 14 and 26°C.

Our final Cox regression model (overall model: χ^2^ = 143.135, *P* < 0.001) included the effect of exposure group (Wald_1_ = 71.712, *P* < 0.001), with *Bd*-exposed frogs having lower survival than sham-exposed animals, and the interaction between bacterial manipulation and temperature (Wald_2_ = 7.453, *P* = 0.024) but not the main effects of temperature (Wald_1_ = 0.122, *P* = 0.727) or bacterial manipulation (Wald_2_ = 3.393, *P* = 0.183) as significant predictors of survival. Considering only the *Bd*-exposed animals, *Bd* load was not a significant predictor of survival (Wald_1_ = 0.153, *P* = 0.695). Frogs in the “*S. maltophilia* added” treatment survived longer than frogs with either “intact” or “reduced” bacterial communities at 26°C (Tarone–Ware: χ^2^ ≥ 5.123, *P* ≤ 0.023). At 14°C, *Bd*-exposed frogs in the “*S. maltophilia* added” treatment survived longer than those in the “intact” bacterial community treatment (Tarone–Ware: χ^2^ = 11.392, *P* ≤ 0.001), but not significantly longer than frogs in the “reduced” bacteria treatment (Tarone–Ware: χ^2^ = 0.017, *P* ≤ 0.896; **Figure 3**). When *S. maltophilia* load replaced bacterial manipulation in our Cox regression model, only the interaction between *S. maltophilia* load and temperature was significant (Wald_1_ = 5.852, *P* = 0.016). Separate Cox regressions for animals at 14 versus 26°C showed that odds of mortality in *Bd*-exposed frogs decreased with increasing *S. maltophilia* load at 14°C (*B* = -0.547, Wald_1_ = 5.731, corrected *P* = 0.034) but at 26°C, *S. maltophilia* load was not a significant predictor of mortality (Wald_1_ = 2.128, corrected *P* = 0.290).

## Discussion

We examined temperature’s effect on the ability of skin microbes to protect a susceptible frog species against *Bd* infection and chytridiomycosis. Given that amphibian immune function is reduced at low temperatures (Rollins-Smith and Woodhams, 2012), and that *Bd* infections and chytridiomycosis are more prevalent in animals with cooler body temperatures (Rowley and Alford, 2013) and in cool climates (Berger et al., 2004; Woodhams and Alford, 2005; Rohr and Raffel, 2010), we predicted that temperature would affect the susceptibility of *A. crepitans* to *Bd* infection and chytridiomycosis in this experiment. Specifically, we predicted that, as in a previous study (Sonn et al., 2017), *Bd*-exposed frogs housed under colder conditions would have higher pathogen loads, a greater likelihood of becoming infected, lower body condition, and/or lower survival. We also predicted that frogs treated with antibiotics prior to *Bd* exposure would be more susceptible, and frogs with the anti-*Bd* bacterium *S. maltophilia* added to their skin would be less susceptible to infection and/or disease than animals with an un-manipulated bacterial community. And finally, we predicted that the effects of *S. maltophilia* on *Bd* susceptibility would be temperature dependent. Some, but not all, of these predictions were upheld.

Temperature had an effect on the susceptibility of *Bd*-exposed frogs, with animals housed at 14°C having a greater likelihood of infection (**Figure 1A**) and also having greater pathogen loads (**Figure 1B** and Supplementary Figures S2C,D) than frogs housed at 26°C. This is consistent with idea that amphibian immune responses are often improved at higher temperatures (Maniero and Carey, 1997; Rollins-Smith and Woodhams, 2012). It is also consistent with the observations that in nature, (1) individual frogs with higher body temperatures are less likely to be infected with *Bd* (Richards-Zawacki, 2010; Rowley and Alford, 2013) and (2) disease variables and risk of *Bd*-related declines often reach a peak in cool seasons and climates (Berger et al., 2004; Woodhams and Alford, 2005; Kriger and Hero, 2006; Longcore et al., 2007; Rohr and Raffel, 2010). This result is also consistent with a recent laboratory exposure study where the susceptibility of *A. crepitans* to chytridiomycosis was found to be inversely related to temperature (Sonn et al., 2017). In contrast to that study, however, the present study showed no significant main effect of temperature on the body condition or survival of *Bd*-exposed animals. There were, however, significant interactions between temperature and bacterial manipulation and between temperature and *S. maltophilia* load for survival, suggesting that the effect of temperature on the survival of *Bd*-exposed frogs depended on their cutaneous bacterial community.

While we predicted we would see significant main effects of bacterial manipulation on several indices of susceptibility to *Bd* infection and chytridiomycosis, the only variable we measured that appears to have been affected by our bacterial manipulations was survival, which differed in a temperature-dependent manner across our bacterial manipulation groups. On average, *Bd*-exposed frogs in the “reduced” bacterial treatment survived equally well at 14 and 26°C whereas frogs with “intact” or “*S. maltophilia* added” bacterial communities survived longer at the higher temperature (**Figure 3**). Frogs in the “*S. maltophilia* added” treatment at 26°C had the greatest mean survival time of all *Bd*-exposed groups.

*Stenotrophomonas maltophilia* has been found not only on frog skin, but also in water, soil, and plant samples from a wide variety of environments and geographic regions (Denton and Kerr, 1998). It is known to inhibit the growth of a broad range of plant (e.g., Elad et al., 1994; Kobayashi et al., 1995; Berg et al., 1996), and even some human (e.g., *Candida* spp., *Aspergillus fumigatus*; Kerr, 1996) fungal pathogens. *Stenotrophomonas* species have been isolated from amphibian skin on several continents (North America: Woodhams et al., 2007b; South America: Flechas et al., 2012; Australia: Woodhams et al., 2015) and *S. maltophilia* (Flechas et al., 2012; Robak, 2016) and some of its congeners (Woodhams et al., 2015) have been shown to inhibit *Bd* growth *in vitro*. We chose *S. maltophilia* for use in this study because our previous work suggested that *S. maltophilia* inhibits *Bd* growth *in vitro* across a range of temperatures (Robak, 2016). However, despite having higher average *S. maltophilia* loads on their skin during this experiment, we did not see a lower probability of infection or lower pathogen load on *Bd*-exposed animals in our “*S. maltophilia* added” treatment, compared with animals in our “intact” bacterial treatment. Importantly though, we did see greater survival in the “*S. maltophilia* added” treatment, compared to our “intact” treatment animals at both 14 and 26°C. This suggests that augmentation of the cutaneous *S. maltophilia* population may yield benefits for *Bd*-exposed frogs across a range of temperatures.

We attempted to reduce the number and diversity of bacteria present on the skins of frogs in our “reduced” bacterial treatment via bath in a cocktail of antibiotics. However, our qPCR assays suggest that animals in our “reduced” treatments maintained as much *S. maltophilia* on their skins as animals in our “*S. maltophilia* added” treatments. It is possible that our antibiotic baths failed to reduce the bacterial communities on the frogs that received them. However, *S. maltophilia* is known to be naturally resistant to many broad-spectrum antibiotics (Denton and Kerr, 1998), so the large populations of this bacterial species on the skins of frogs in our “reduced” treatments could also be explained by the growth of *S. maltophilia* (and potentially other antibiotic-resistant bacteria) after the removal of their more antibiotic-susceptible competitors. The lower concentrations of *S. maltophilia* maintained by frogs with putatively more diverse skin communities in our “intact” treatments are consistent with this explanation. If *S. maltophilia* does indeed augment the host’s ability to tolerate a heavy *Bd* infection, this could explain why frogs in our “reduced” treatment, which maintained high *S. maltophilia* loads on their skin, especially at 14°C, survived as long as frogs in our “*S. maltophilia* added” treatment at that temperature. However, in that case, it is not clear why our “reduced” treatment frogs did not receive the same survival benefit as our “*S. maltophilia* added” frogs at 26°C.

At 14°C, but not at 26°C, frogs with abundant *S. maltophilia* on their skin survived repeated exposures to *Bd* longer than frogs with “intact” skin bacterial communities where *S. maltophilia* was present, but less abundant. Interestingly, though *S. maltophilia* (Denton and Kerr, 1998) and other bacteria isolated from *A. crepitans* grow faster *in vitro* at 26 than at 14°C (Robak, 2016), frogs in all three of our bacterial treatment groups maintained more *S. maltophilia* on their skin at 14 than at 26°C (Supplementary Figure S3). It is not clear what caused this difference between *in vitro* and *in vivo* growth of *S. maltophilia* or whether the pattern holds for other members of the microbial community on amphibian skin. Interestingly, the temperature optimal for *Bd* growth on *A. crepitans* (Sonn et al., 2017) and other amphibian hosts (Cohen et al., 2017) also commonly differs from that of growth in culture.

While augmentation with a known anti-*Bd* bacterium was associated with longer survival in *Bd*-exposed animals at both 14 and 26°C, our results also support our prediction of temperature-dependent effects of beneficial skin microbes. For example, at 14°C, there was a positive relationship between *S. maltophilia* load and the odds of survival in *Bd*-exposed frogs. At this temperature, frogs in the “*S. maltophilia* added” and “reduced” bacterial community treatments, which had greater *S. maltophilia* loads (Supplementary Figure S3), survived *Bd* infections longer than did animals in the “intact” bacterial community treatment (**Figure 3** and Supplementary Figure S2E). We observed this difference in survival, despite the fact that *Bd* loads were similarly high among animals in all three bacterial treatment groups, suggesting that at 14°C, the load of *S. maltophilia* on the skin affected the animals’ ability to survive with (i.e., tolerate) a heavy *Bd* infection. In contrast, at 26°C, the relationship between *S. maltophilia* load and survival was not significant.

The mechanism by which tolerance of *Bd* infection is modulated by *S. maltophilia* remains unclear. This bacterium’s inhibition of phytopathogenic fungal growth has been linked to its production the antifungal secondary metabolites pyrrolnitrin (Kerr, 1996) and maltophilin (Jakobi et al., 1996). Interestingly, *S. maltophilia* also exhibits chitinolytic activity (Kobayashi et al., 1995). Chitin is an important part of cell wall structure stability for *Bd* and other chytrid fungi, and drugs that interfere with chitin synthesis have been shown to inhibit *Bd* growth *in vitro* (Holden et al., 2014). It seems likely that this chytinolytic activity plays a role in the effect that heavy loads of *S. maltophilia* on the skin had on the survival of our *Bd*-exposed hosts. At 14°C, *S. maltophilia* load was positively associated with survival in our *Bd*-exposed frogs. However, at 26°C, we saw no significant relationship between *S. maltophilia* load and survival. This could be because the *S. maltophilia* loads on our 26°C frogs never reached the levels that they did on animals at 14°C. Perhaps some threshold load of *S. maltophilia* is needed before the benefits of this microbe can be seen and the higher temperature prevented frogs in our 26°C treatments from reaching this threshold? It could also be that heavy *Bd* infections facilitate frogs sustaining large populations of *S. maltophilia* on the skin, as we found a positive relationship between *Bd* load and *S. maltophilia* load at 14°C (**Figure 2**). In this case, the lack of an effect of *S. maltophilia* on the survival of *Bd*-exposed animals at 26°C could be explained by the lower average *Bd* loads these animals experienced (**Figure 1B**).

All but one of the *Bd*-exposed frogs in our 14°C treatments became heavily infected with *Bd* during our experiment and most died after exhibiting clinical signs of chytridiomycosis. Manipulation of the bacterial community appears to have affected the survival time of heavily infected frogs (i.e., tolerance) but not the likelihood of infection (i.e., resistance). However, the infections we observed in *Bd*-exposed frogs at 26°C were generally light and often transient. In many cases, death at this temperature was preceded by one or more weekly skin swabs that tested negative for the presence of *Bd*. It seems unlikely that these animals at 26°C were dying of chytridiomycosis, though they exhibited similar clinical signs to animals that died with high *Bd* loads in the 14°C treatments. However, survival of the *Bd*-exposed frogs was significantly lower than sham-exposed animals at both temperatures, suggesting that mortality was due to *Bd* exposure and not another pathogen or husbandry-related cause.

While it is not uncommon in *Bd* exposure studies for hosts to remain uninfected or clear *Bd* infections (e.g., Ramsey et al., 2010; Brannelly et al., 2012), we are not aware of other published studies where high mortality was seen in animals with transient and generally low-intensity *Bd* infections. We can think of two plausible explanations for the mortality experienced by our *Bd*-exposed animals at 26°C, both of which are related to our having exposed these animals repeatedly to high concentrations of this pathogen.

First, resisting infection can be costly (Dallas et al., 2016), especially if it involves activation of the immune system and/or stress response. Given that amphibian immune function is often temperature dependent (Rollins-Smith and Woodhams, 2012), the cost of resisting infection may depend upon temperature as well. Though this topic remains understudied, evidence for a cost of resisting *Bd* infection exists for newts (Cheatsazan et al., 2013) and tadpoles (Gabor et al., 2017). If such a cost exists for *A. crepitans*, it could explain the mortality we saw in *Bd*-exposed frogs at 26°C, though in that case, it is perhaps surprising that we did not see a decline in body condition in these animals. On the contrary, both *Bd*- and sham-exposed frogs at 26°C gained body condition over the course of the study and at no point in the experiment, there was a difference in body condition between these two exposure groups (Tukey HSD: *t*_672_ ≤ 1.895, *P* ≥ 0.058).

Second, *Bd* is known to produce and release toxic factors that cause pathology and mortality in crayfish, even in the absence of infection (McMahon et al., 2013). *Bd* is also known to produce a toxic factor or factors that inhibit immune responses to *Bd in vitro* (Fites et al., 2014) and possibly also *in vivo* (Ellison et al., 2014). While it is not clear whether the pathology and mortality in crayfish and immune inhibition in amphibians are generated by the same or different toxic factors, it seems likely that this fungus, like many others (Bondy and Pestka, 2000), produces toxins capable of affecting the fitness of amphibian hosts, perhaps even in the absence of an active infection in the skin (e.g., in tadpoles: Blaustein et al., 2005). While we cannot definitively attribute the mortality seen in our 26°C treatment to a toxin, if *Bd* does produce a substance capable of causing mortality in amphibians, our methods may have been more likely to produce this effect than the methods of other similar studies. We exposed frogs weekly by bath to small volumes of water containing millions of zoospores whereas other studies have tended to use fewer exposures and lower concentrations of *Bd* (e.g., reviewed in Kilpatrick et al., 2009). Not much is known about the frequency of exposure or the concentration of *Bd* in natural environments, so it is unclear whether our results would be expected to hold in the wild. However, a mark-recapture study of Louisiana *A. crepitans* suggests that repeated exposure and cycles of clearance and re-infection are common (Brannelly et al., unpublished data). We suggest that the potential for mortality due to toxin exposure rather than skin infection in amphibians exposed to *Bd* deserves further study.

Our results demonstrate that both temperature and the makeup of the skin bacterial community can impact the susceptibility of amphibian hosts to chytridiomycosis. Temperature’s main effects were on the likelihood (i.e., resistance) and magnitude of infection whereas the skin microbial community affected the host’s ability to survive a heavy infection (i.e., tolerance). Frogs at 14°C survived longer, despite large *Bd* burdens, when they harbored large populations of the antifungal bacterium *S. maltophilia* on their skin. Survival of frogs with *S. maltophilia*-enhanced skin communities was also longer at 26°C, though at this temperature, survival was not correlated with *S. maltophilia* load and exposure to, rather than infection with, *Bd* seems to have been the main cause of mortality. Whether this sort of interaction between temperature and the protection that bacteria provide against animal pathogens is common remains to be seen. Given its importance to the physiology of all three players, we predict that temperature may have especially strong impacts on the interactions of ectotherm hosts and their bacterial communities with fungal pathogens (Fisher et al., 2012; Carey and Duddleston, 2014; Daskin et al., 2014).

## Author Contributions

MR and CR-Z designed the study. MR conducted the lab work and wrote the first draft of the manuscript. CR-Z revised it for publication. Both authors analyzed and interpreted the data and approved the manuscript’s content.

## Conflict of Interest Statement

The authors declare that the research was conducted in the absence of any commercial or financial relationships that could be construed as a potential conflict of interest.
