# Supplementary material for: Temperature-Dependent Effects of Cutaneous Bacteria on a Frog’s Tolerance of Fungal Infection
Source: Front Microbiol. 2018 Mar 7;9:410. doi: 10.3389/fmicb.2018.00410 (PMC5845872; doi:10.3389/fmicb.2018.00410)
Supplement: Supplementary file 1 [file Image_1.pdf]

Online Supplementary Material

Temperature dependent effects of cutaneous bacteria on a frog's tolerance of *Batrachochytrium dendrobatidis* infection

Matthew J. Robak and Corinne L. Richards-Zawacki

Supplementary Figures

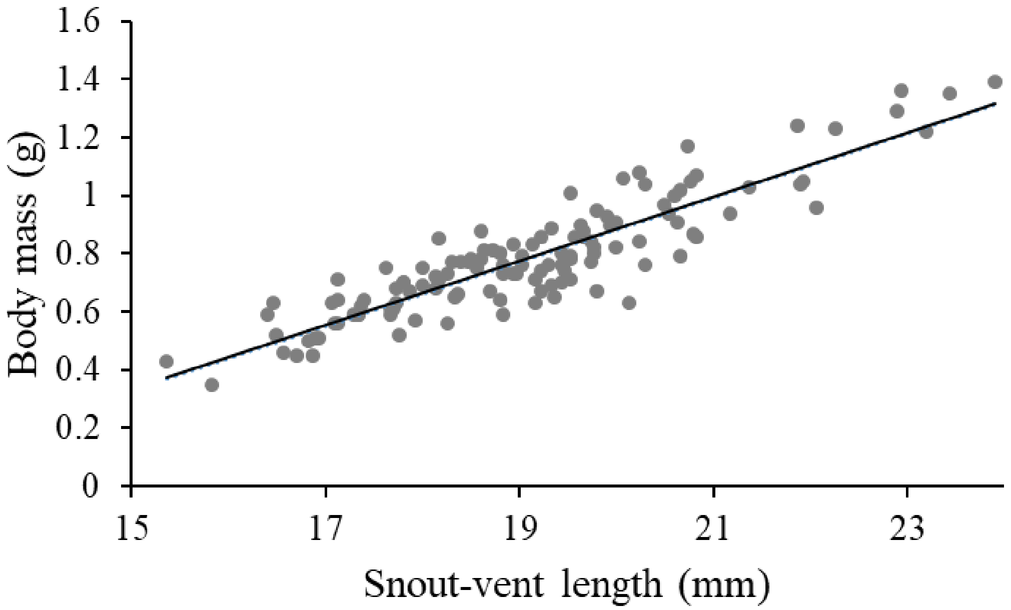

Figure S1. Relationship between snout-vent length (SVL, in mm) and body mass (g) for frogs on day -1 of the experiment. Line is the least-squares line of best fit ($y = 0.1105x – 1.3251$; $R^2 = 0.8504$). No outliers were present in the dataset.

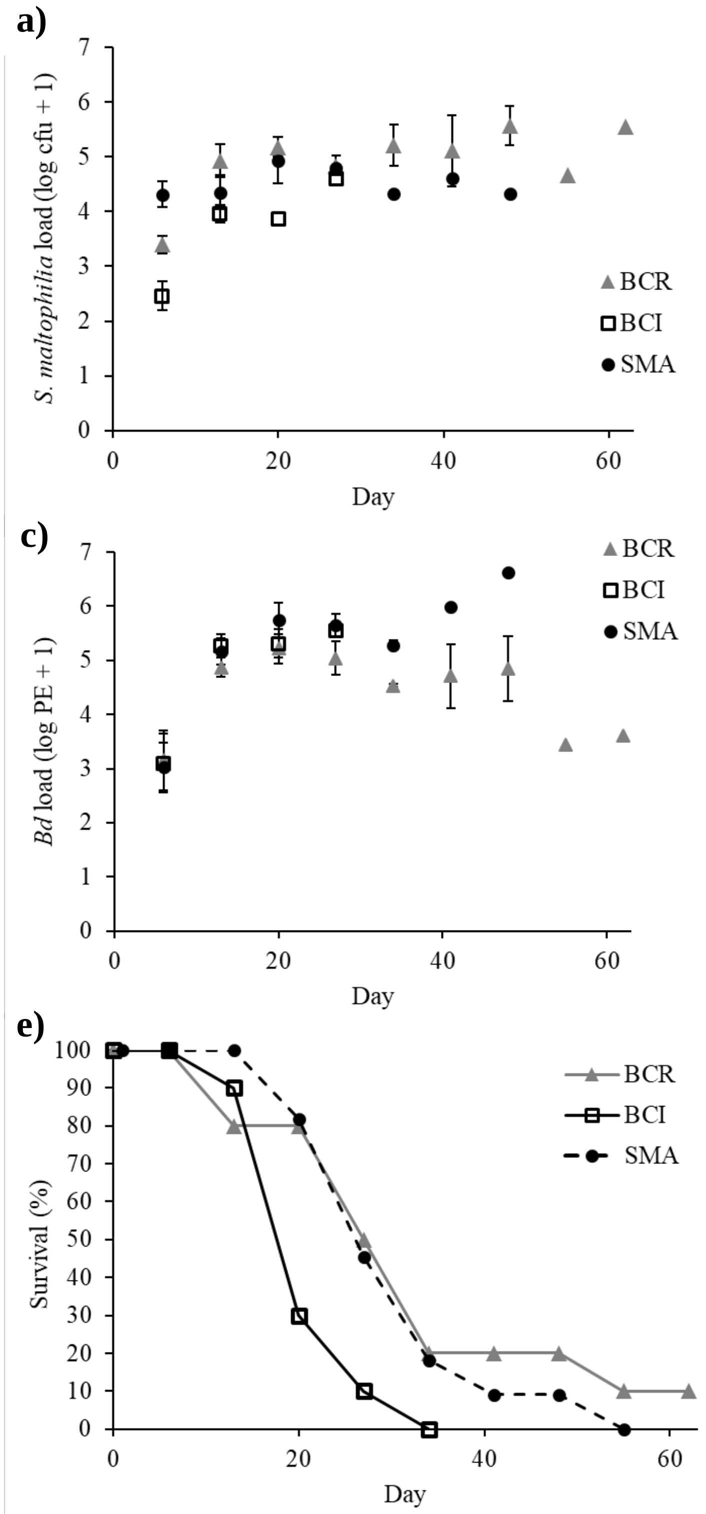

Figure S2. Weekly (**a** and **b**) *S. maltophilia* loads (measured in log_{10} cfus) and (**c** and **d**) *Bd* loads (measured in log10 PE) and (**e** and **f**) survival (%) for *Bd*-exposed frogs in each bacterial manipulation (BCR = bacterial community reduced, BCI = bacterial community intact, SMA = *S. maltophilia* added) at 14 °C (**a**, **c** and **e**) and 26 °C (**b**, **d** and **f**). Error bars are ± 1 SE of the mean.

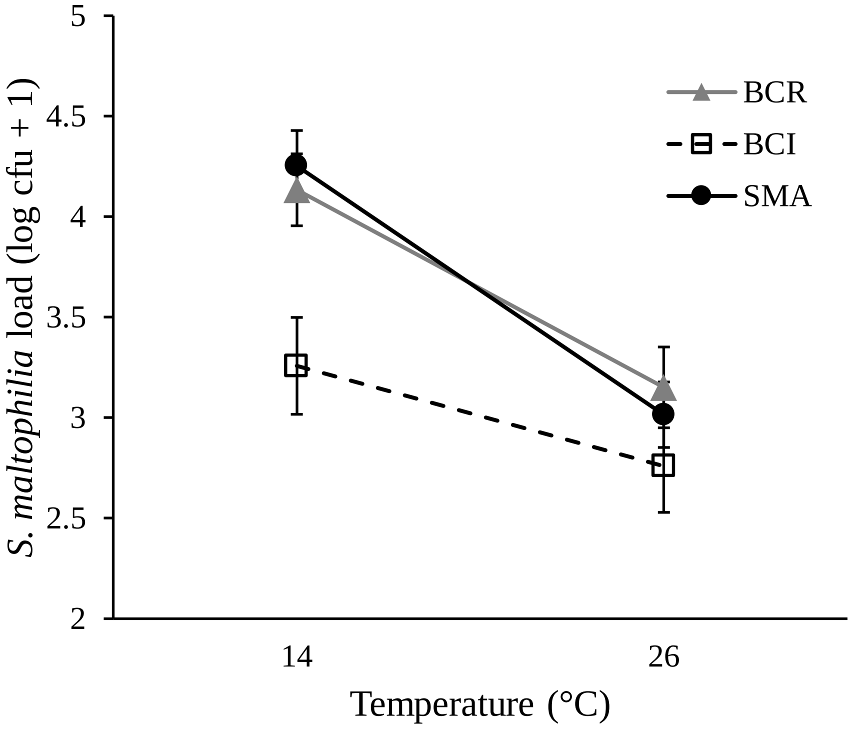

Figure S3. *S. maltophilia* load (mean $\log_{10}$ ± SE of cfus) for *Bd*-exposed frogs by temperature and bacterial manipulation (BCR = bacterial community reduced, BCI = bacterial community intact, SMA = *S.maltophilia* added).

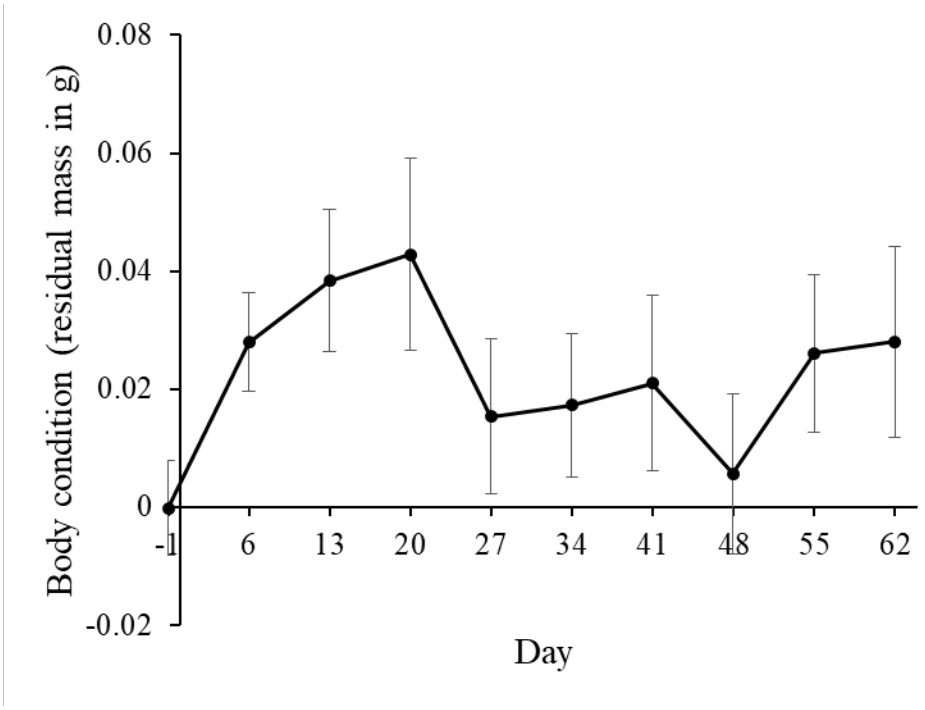

Figure S4. Body condition (mean ± SE), expressed as residual mass with respect to a regression of mass on snout-vent length at day -1, for frogs in all treatment groups over the course of the experiment. The only significant changes in body condition were between day -1 and days 6, 13, and 20.
